# Potential impact of subsequent entry biologics in nephrology practice in Canada

**DOI:** 10.1186/s40697-014-0032-7

**Published:** 2014-12-19

**Authors:** Daniel J Martinusen, Clifford Lo, Judith G Marin, Nicole W Tsao, Marianna Leung

**Affiliations:** British Columbia Provincial Renal Agency, Vancouver, Canada; St. Paul’s Hospital, Providence Healthcare, Vancouver, Canada; Faculty of Pharmaceutical Sciences, University of British Columbia, Vancouver, Canada; Royal Jubilee Hospital, Island Health Authority, 1952 Bay Street, Victoria, British Columbia V8R 1J8 Canada

**Keywords:** Subsequent entry biologics, Biosimilars, Biotherapeutic products, Biologics, Nephrology, Regulation

## Abstract

**Purpose of review:**

Subsequent entry biologics may soon be a reality in Canadian nephrology practice. Along with opportunities to reduce health care costs, these agents pose unique challenges that must be met for successful implementation. Understanding the experiences around the globe in both regulatory affairs and implementation will be a valuable guide for Canadian clinicians. This report provides an executive summary of the information required to guide decisions to use or implement subsequent entry biologics by comparing Canadian regulations to other developed nations, discussing their clinical issues and predicting their impact on the Canadian market and nephrology practice. We hope that this review will assist clinicians and policy makers to navigate this complex subject and to make informed decisions in the best interest of their patients.

**Sources of information:**

Sources of information include published literature and reports available in the public domain including guidelines obtained from regulatory agencies and information shared by Pharmaceutical companies. Lastly, we generated information from our own focus group consisting of nephrologists, a regulatory body representative, a hospital formulary representative, a patient representative, a hospital administrator, and a health economist.

**Findings:**

There exists a common and robust approach in the G20 countries for approval and regulation of subsequent entry biologics. Although by definition these agents do not have advantages (other than costs) or disadvantages compared to the original biologic, there are potential concerns and economic uncertainties regarding their implementation. Where SEBs are on the market, their market share is variable and modest.

**Limitations:**

We did not purchase third party reports for up to the minute marketing data. Since there are no subsequent entry biologics currently on the Canadian market, the information is only predictive.

**Implications:**

The nephrology community will have to work with patients, payers, and regulatory bodies to ensure safe and effective use of subsequent entry biologics. Cost savings can be achieved but these agents should only be used after fully understanding their unique challenges. At this time, they should not be automatically substitutable and only used for Health Canada-approved indications. Only through good pharmacovigilence will health care providers and patients become better informed.

## ABRÉGÉ

**Objectif de la revue:**

L’utilisation des produits biologiques ultérieurs pourrait bientôt devenir réalité en néphrologie au Canada. Ces agents, qui permettent possiblement des réductions de coûts en soins de santé, présentent des défis particuliers qui doivent être évalués pour une introduction réussie sur le marché. À cet effet, arriver à comprendre les différentes expériences qui se sont déroulées partout dans le monde en matière de réglementation et de mise en œuvre de ces agents est un exercice précieux qui pourrait guider les praticiens canadiens. Ce rapport fournit un résumé des informations requises pour guider la prise de décision pour l’utilisation et l’introduction des produits biologiques ultérieurs : comparaison des réglementations canadiennes à celles d’autres pays; discussion des enjeux cliniques entourant les produits biologiques ultérieurs et prévision de l’impact de leur utilisation sur le marché canadien et sur la pratique de la néphrologie. Nous espérons que cette revue aidera les cliniciens et les décideurs dans l’exploration de ce sujet complexe et dans une prise de décision optimale pour leurs patients.

**Sources d’information:**

Plusieurs sources d’information ont été utilisées, dont des publications et des rapports du domaine public, sous la forme de lignes directrices d’organismes de réglementation et de documents provenant de compagnies pharmaceutiques. De plus, nous avons utilisé l’information générée par notre propre groupe de discussion. Ce dernier est composé de néphrologues, d’un représentant d’organisme de réglementation, d’un représentant de la Liste des médicaments des hôpitaux, un représentant des patients, d’un directeur d’hôpital et d’un économiste de la santé.

**Résultats:**

Dans les pays membres du G20, il existe une approche robuste communément adoptée pour l’approbation et la réglementation des produits biologiques ultérieurs. Par définition, ces agents ne présentent aucun avantage (sauf économique),ou désavantage particulier lorsqu’on les compare aux produits biologiques originaux, mais certaines préoccupations persistent quant à leur introduction sur le marché. Par ailleurs, lorsque les produits biologiques ultérieurs sont disponibles sur le marché, leur part de marché reste modeste et variable.

**Limites de l’étude:**

Nous n’avons pas fait l’acquisition de rapports en provenance de tiers contenant des données de commercialisation récentes. De plus, puisqu’il n’existe pas de produits biologiques ultérieurs sur le marché canadien à l’heure actuelle, l’information de cette revue n’est que de nature prédictive.

**Implications:**

L’ensemble des néphrologues auront à collaborer avec les organismes de régulation, les patients, et les contribuables afin d’assurer une utilisation efficace des produits biologiques ultérieurs. Des économies peuvent être réalisées, mais ces agents ne devront être utilisés qu’après avoir tout à fait compris les défis particuliers qu’ils présentent. En ce moment, ils ne peuvent pas remplacer automatiquement d’autres produits et ne devraient être utilisés que selon les recommandations officielles de Santé Canada. Ce n’est qu’en exerçant une bonne pharmacovigilance que les patients et prestataires de soins de santé deviendront mieux informés.

## What was known before

Epoetin biosimilars (also known as subsequent entry biologics in Canada) have been marketed in Europe since 2007 and will enter the Canadian market in the near future. The reason for using these agents is their reduced cost.

## What this adds

This paper provides an executive summary of the information required to guide decisions to use or implement subsequent entry biologics by comparing Canadian regulations to other developed nations, discussing their clinical issues and predicting their impact on the Canadian market and nephrology practice.

## Why this report is important

The Canadian nephrology community will have to make decisions regarding to use of subsequent entry biologics in the near term, these decisions will determine the allocation of hundreds of millions of dollars. This report should assist in making these important decisions.

## Key messages

Subsequent entry biologics should not be thought of as generic versions of the innovator biologic and therefore decisions to use them are not straightforward. Unlike generic medications, Health Canada considers subsequent entry biologics to be not interchangeable or substitutable with the innovator product. The use of subsequent entry biologics should be a therapeutic decision made in consultation with a physician.

## Implications for future research/policy

In addition to financial variables, key policy decisions should take into consideration physician, pharmacist and patient preferences such as those that can be elicited through discrete choice experiments. Policies influence nephrology should also be made in partnership with other medical specialties who use subsequent entry biologics, with all parties cognizant of their impact on the majority payer and to make decisions that will result in the greatest benefit to the health care system.

## Introduction

Biologic medicines have contributed to the health of Canadians since the 1980s and the offerings of these complex therapies have expanded greatly. Originally consisting of proteins of varying lengths, biologics introduced recently tend to be very large monoclonal antibodies with very specific targets. These innovations to health, while welcomed, have come at great financial expense. As the patents expire for many of these products within this decade, there are opportunities for a competitive marketplace to reduce these costs by marketing “copies” of innovator biologics called subsequent entry biologics (SEBs). In Canada, SEBs are defined by Health Canada as biologics that are similar to, and would enter the market subsequent to, an approved innovator biologic. However, unlike small-molecule generic medicines, production of biological molecules is complex and exact copies cannot be produced. SEBs are derived by complex living systems and are comparatively much larger molecules than the traditional small molecules: aspirin, for instance, has a molecular weight of 180 daltons [1], compared with erythropoietin at 30,400 daltons [2], and monoclonal antibodies at 150,000 daltons [3].

The manufacture of small molecules follows a well-defined and reproducible chemical synthesis for which the resulting product can be easily compared to the innovator with about 50 tests [4]. By comparison, the SEB is the result of a carefully and tightly controlled biological process where there may be as many as 200 complex comparative tests [4,5]. Given that these proteins are derived from complex living organisms, the resulting structure can never be identical to the innovator or “reference” biologic. Therefore, it is important to strike the right balance between maintaining the safety and effectiveness Canadians expect and deserve, reducing costs and encouraging innovation for better therapies.

This paper serves as an executive summary of the work undertaken by an independent group of pharmacists, at the request of the Canadian Society of Nephrology, to evaluate the impact of SEBs on Canadian nephrology practice. A full text of the original report is available on the Canadian Society of Nephrology website, and this paper serves as an executive summary of the project, discussing main issues and key recommendations. In addition, a full systematic review of safety and efficacy as well as an economic analysis of epoetin SEBs in Canada was undertaken; these are presented in separate publications and also in the full report.

In order to develop an approach to SEBs, we undertook an extensive environmental scan, including an understanding of the current (2013 to 2014) worldwide use of SEBs as well as national and international regulations and policies. Using this multi-pronged approach, we are able to present for the nephrology community, a synthesis of key points required to understand the issues, as well as recommendations for moving forward as a responsible community of care providers in nephrology.

## Review

### Clinical issues regarding SEBs

As a concept, SEBs have no advantage (other than cost) or disadvantage over the reference biologic. Nonetheless, their introduction to the market place will have a number of benefits linked to other positive outcomes. As the price of biologics fall, more patients may be offered the treatment and/or the treatment may be offered earlier in the course of the disease. In other contexts where expanded use of an agent may not be applicable, for example with respect to erythropoiesis stimulating agents (ESAs) for the treatment of anemia of chronic kidney disease (CKD), reduced acquisition costs will be realized by the health payer (increase efficiency), and may be used to fund other aspects of care. Increased competition in Canada may incent provisions of additional value, such as making better delivery devices, training, education and other support services for patients and caregivers. System-wide, they may improve reimbursement and third-party payer support in addition to the education and clinical data needed by clinicians.

Another potential benefit will be the drive to innovate (to retain market share) by developing second or third generation biologics. Innovator companies can extend the half-life of a biologic, increase potency, reduce toxicity and immunogenicity or improve the method of administration. Humanization of biologics will likely be an important goal to further improve current offerings. For anemia, novel erythropoietin receptor agonists are being developed. Companies will be invested in this development as regulators will extend the patent life on the drug or if deemed a new drug, the company will get significant market exclusivity. Over the long term, therapies for Canadians will continue to improve.

However, SEBs are not without risks. Immunological reactions to a biologic drug are amongst the most important adverse reactions that are possible. While reactions can be simply developing antibodies with no clinical consequences, antibodies themselves can result in systemic immune reactions, autoimmunity to endogenous proteins or the undermining of therapeutic efficacy through antibodies targeting endogenous proteins [6]. Nephrologists are well aware of PRCA that occurred as a result of a formulation change of Eprex® introduced years after original marketing, despite the comparability exercises performed. The manufacturer changed the processing of the rubber in the prefilled syringe and the formation of polysorbate 80 micelles containing proteins. This then influenced the formation of aggregates/impurities and resulted in increased immunogenicity expressed as pure red cell aplasia (PRCA) [7]. Hence, a product in use for years with a good safety record was unfortunately responsible for serious side effects, due to a change in the manufacturing process. Similar concerns were raised for the biosimilar epoetin HX575 in trial data and it remains indicated for only the intravenous route, however studies on this are ongoing. Our independent systematic review and meta-analysis found little difference in safety and efficacy between epoetin SEBs and their reference biologic. Yet, PRCA highlight the importance of comparability studies as well as strong pharmacovigilence programs [8].

Biologic drug manufacturers over time have made changes to the way each biologic is manufactured to improve efficiency or quality or both [9]. With each change, the resulting product must be compared to the molecule before the change. The manufacturer works with the national regulatory body to conduct an internationally standardized comparability exercise to ensure that the change has not adversely affected the product safety or efficacy [10,11]. Testing for immunogenicity is an important aspect of comparing an SEB to a reference product as well as comparing a biologic pre- and post-manufacturing change. These PRCA incidents warrant a better understanding of PRCA pharmacoepidemiology, the risk chain of anti-erythropoietin antibody development, consistency in the assay used, a validated serum-cut-off value and differentiation between neutralizing antibody to PRCA development [7]. Health Canada directs that to study the safety and efficacy of the product, validated methods should be used to characterize the antibody content (concentration or titre) and the types of antibodies (neutralizing or cross reacting) [12]. Unfortunately such a change may not be detected until it has widespread use.

The comparability exercises for a manufacturing change to an originator biologic forms the basis for the regulatory framework of SEBs to ensure they are safe and effective. The goal is to safely bring a similar enough biologic to market at a reduced cost to both the manufacturer and the consumer while maintaining efficacy.

## Regulatory agencies and international approaches

The European Medicines Agency (EMA) was the first to create a legal pathway for SEBs in 2005, followed by guidelines for approval in 2006. Since then, Australia, Brazil, Canada, Cuba, India, Japan, Malaysia, Singapore, South Korea, Taiwan, Turkey, United States and the World Health Organization have all created a regulatory framework for SEBs. Guidelines around the world are becoming similar and strike a balance between scientific comparability, sound safety and efficacy testing while creating a less costly pathway to approval.

China, Russia and Thailand are the exceptions. China has had no biosimilar pathway and has treated each submission as a new drug. China is actively working toward a biosimilar pathway and has indicated that the creation of a strong biologic sector is one area of focus over the coming years.

A comprehensive review of the different world wide regulatory approaches is available in our full report; the following summarizes the leading regulatory frameworks that exist. The reader should be aware that SEBs have different names in other countries such as biosimilars according to the European Medicines Agency, or similar biotherapeutic product according to the World Health Organization. Despite differences in nomenclature, similar principals apply as can be seen in Table 1.

**Table 1:** **Comparison of requirements for the evaluation of SEBs between different regions** [13]

	**EU**	**Australia**	**Japan**	**WHO**	**Canada**	**Korea, India, Singapore, Malaysia**
**Synonym**	Biosimilars	Biosimilars	Follow-on Biologics	Similar Biotherapeutic Product	Subsequent Entry Biologic	Biosimilars
**Scope**	Mainly recombinant protein drugs		Recombinant protein drugs			
**Principles**	• Generic approach is not appropriate for SEB.					
	• SEB should be similar to the reference biologic with respect to quality, safety and efficacy.					
	• Step-wise comparability approach: the reduction of non-clinical and clinical data required will only be considered after the similarity of the SEB and reference biologic is proven in terms of quality.					
	• Case by case approach for different classes of products.					
	• Pharmacovigilance is stressed.					
**Reference product**	Authorized in the EU		Authorized in Japan	Authorized in a region with a well-established regulatory framework		
**Manufacture**	• Same standards required by the national regulatory agency for originator products.					
	• Full chemistry and manufacture data package.					
**Physio-chemical**	• Primary and higher-order structure.					
	• Post translational modifications.					
**Purity**	• Process-related and product-related impurities.					
**Non clinical**	• In-vitro such as cell-based assays and receptor-binding studies.					
**Stability**	Accelerated degradation studies and studied under various stress conditions		Not necessary	Accelerated degradation studies and studied under various stress conditions		
**Pharmacokinetic study design & criteria**	• Single dose, steady-state studies or repeated determinations of pharmacokinetics					
	• Cross over or parallel.					
	• Include absorption and elimination characteristics.					
	• Use the traditional 80-125% equivalence range.					
**PD**	Pharmacodynamic (PD) markers should be selected and comparative PK/PD studies may be appropriate					
**Efficacy**	Comparability margins should be pre-specified and justified			Observer or double-blinded. Equivalence or non-inferiority		Equivalence
**Safety/pharmacovigilance**	• Pre-licensing safety data and risk management plan. Post authorization safety and/or efficacy studies may be required.					
	• Adverse reactions must be reported.					
	• Same rules apply to reference biologic and SEB.					
**INN vs. new generic name**	INN	Unique generic name	Suffix “BS” added to INN	INN with biological qualifier (proposed)	INN but will follow WHO guidance	INN
**Extrapolation of indications**	• Assessed on a case-by-case basis.					
	• At least one clinical study required in the most sensitive population measuring the clinical endpoints likely to show a difference.					

Adapted from [13].

### Health Canada

The approach to SEBs taken by Health Canada is consistent with that of the EMA and the WHO; sponsors are even referred to the EMA product class specific guidance documents (for instance, epoetin, G-CSF and growth hormone) because of the similarities in scientific principles [14].

SEBs are approved through the New Drug Submission pathway in Canada. This pathway requires the SEB sponsor to submit a full chemistry and manufacturing data package in addition to extensive data demonstrating similarity of the SEB with the reference biologic. This includes characterization studies conducted in a side-by-side format to determine physiochemical properties, biological activity, immunochemical properties, purity, impurities, contaminants, and quantity [12]. The demonstration of similarity does not signify that the quality attributes of the SEB and the reference biologic being compared is identical, but that they are highly similar with the following two consequences [12]:

1. The existing knowledge of both products is sufficient to predict that any differences in quality attributes should have no adverse impact upon safety or efficacy of the SEB.
2. Non-clinical and clinical data previously generated with the reference biologic drug are relevant to the SEB.

If similarity of an SEB to the reference biologic drug cannot be established based on the chemistry and manufacturing data package, then reduced clinical data cannot be justified and the product cannot be considered as an SEB [12].

Any claims made by the SEB sponsor should be supported by suitable scientific data, which typically includes safety and efficacy data as well as comparative pharmacokinetic (PK)/pharmacodynamic (PD) studies [12]. As for efficacy and safety studies, head-to-head equivalence trial(s) are preferred as they define upper and lower comparability margins. The regulatory agency may accept non-inferiority trials, which describe only the lower efficacy margin. Studies may not be deemed to provide strong enough support for extrapolation to other indications approved for the reference biologic, particularly if the other indications include different dosages than those tested [12]. However, additional indications may be granted to the SEB in the absence of clinical data with only comparative PK/PD data and in certain cases it may be possible to extrapolate clinical data to other indications where rationales are sufficiently persuasive [12]. The decision is based on mechanism of action, disease pathophysiology, safety profile and clinical experience [12]. Recently, Health Canada granted limited extrapolation of indications to the SEB infliximab [15]. Of the 50 countries granting approval to the biosimilar infliximab, only Japan and Canada limited the indications compared to the innovator drug.

Although SEBs must demonstrate similarity to the reference biologic, they are approved as a new drug and therefore they are not declared to be pharmaceutically or therapeutically equivalent to their reference biologic according to Health Canada [14]. Unfortunately, specialized studies supporting therapeutic interchange are not usually done and their relevance may not be long lasting due to manufacturing changes over time [14]. It is for this reason that Health Canada does not support automatic substitution and recommends physicians make well informed decisions regarding therapeutic interchange [14]. Admittedly, the authority to declare two products automatically substitutable by a pharmacist does not rest with the federal government, but Health Canada’s position and regulatory process should inform decisions regarding interchangeability and substitutability [14].

Recognizing Canada represents a small portion of the global market share for biologic medications, Health Canada has taken the WHO position that differs from the EMA, the United States FDA, the Australian Therapeutic Goods Administration and the Japanese Pharmaceutical and Medical Devices Agency, by allowing the “reference biologic” (to which the SEB is compared) to be a product that is not authorized for sale in Canada. If a non-Canadian reference biologic is used, certain criteria must be met such as [12]:

- The sponsor is responsible for showing that the non-Canadian reference biologic is a suitable proxy for the version of product approved in Canada.
- The sponsor has the responsibility of ensuring that the chosen non-Canadian reference biologic drug has associated with it sufficient information and data to support the submission
- The non-Canadian reference biologic drug is from a jurisdiction that has an established relationship with Health Canada.
- The non-Canadian reference biologic is widely marketed in a jurisdiction that formally adopts International Conference on Harmonization guidelines and has regulatory standards and principles for evaluation of medicines, post-marketing surveillance activities and approach to comparability that are similar to Canada.
- If the non-Canadian reference biologic is used in clinical studies in Canada, data must be provided to satisfy chemistry and manufacturing information as per C.05.005 of the *Food and Drug Regulations.*

Lastly, the SEB sponsor must develop, maintain and implement a risk management plan (RMP) which provides proposals on how to minimize any identified or potential safety risks throughout the life cycle of the product as well as provide a pharmacovigilance plan which identifies and characterizes known or potential safety concerns. In Canada, market authorization holders have primary responsibility for the safety of any products they sell, manufacture, import or distribute to the Canadian public [16]. It is mandatory for market authorization holders to report adverse drug reactions (ADRs), notify the Minister of Health of a significant change in a product’s benefit-risk profile and provide an overall safety evaluation of the product in the form of an annual summary report [16]. Consumers, patients and health professionals are also encouraged to report adverse reactions through MedEffect™ Canada [16]. Lastly, Health Canada is committed to aligning with international best practices and standards including commitment to full integration of International Conference on Harmonization vigilance tools [16].

### World Health Organization

The WHO has served to harmonize the biologic medicine regulation worldwide and is well placed to do so for biosimilars. In seeking common ground amongst countries, it must also allow for variability where there is currently debate. Hence, while the EMA is quite specific, the WHO guidelines are replete with “should” statements in comparison. That being said, the WHO did rely heavily on the EMA guidelines to create an international standard since the EMA has the most advanced regulations.

Like the EMA, the WHO shares the key principles of a stepwise approach to determine the quality attributes of a product followed by non-clinical and clinical studies. The quality studies need to demonstrate a consistent and robust production, a complete characterization of the product and a complete comparability exercise. Only then will a regulator consider a reduced data requirement for the non-clinical and clinical development. Finally, the amount of data in the non-clinical and clinical portions of the submission is dependent on the therapeutic class of biologic and so considered on a case-by-case approach.

The WHO guidelines recognize that a reference product for comparison may not be marketed in all countries, particularly those with a small share of the global market. Unlike the EMA, the WHO allows for a reference product to be used that is marketed outside the jurisdiction of the national regulatory agency. The reference should have been marketed for a sufficient amount of time and in significant volume as well as having been licensed on full quality, safety and efficacy data. Additionally, the WHO, like the EMA, directs that the same reference should be used throughout the comparison and that the SEB should be the same in substance, dosage form and route of administration as the reference.

Good Manufacturing Practices should be implemented and the “similar biotherapeutic product” should meet the same manufacturing standards of the national regulatory agency as the reference drug. All aspects of the manufacturing process should form part of the submission. Additionally, characterization studies including higher order structure, post-translational modification, biological activity, impurities, immunogenicity, accelerated degradation studies and studies under various stress conditions should be included.

Non-clinical studies should be in-vitro (such as receptor binding) and in-vivo (such as repeat dose toxicity studies or product neutralizing capacity), consistent with the EMA guidelines. The clinical studies begin with the PK and PD followed by the clinic trials for efficacy. PK equivalence between the similar biotherapeutic product and the reference product is proven if it falls between 80-125%. The clinical trials should be randomized; well controlled and double-blinded or at least investigator-blinded [13]. Equivalence trials are preferred as they define upper and lower comparability margins. If justified, the regulatory agency may accept non-inferiority trials, which describe only the lower (efficacy) margin. These clinical trials should also produce the pre-licensing and immunogenicity data for the submission.

Similar biotherapeutic product manufacturers must submit a risk management plan and pharmacovigilance plan. It is recognized that the limited exposure in a clinical trial may not fully characterize the adverse events that may be experienced when marketed. With this in mind, post-marketing monitoring is an essential component to ensure ongoing safe use. Lastly, the WHO states that interchangeability is a national decision and beyond the scope of the WHO guidance document.

### European Medicines Agency

In Europe, a biosimilar is a biological medicinal product that contains a version of the active substance of an already authorized original biological medicinal product (reference medicinal product). A biosimilar demonstrates similarity to the reference medicinal product in terms of quality characteristics, biological activity, safety and efficacy based on a comprehensive comparability exercise [17].

In 2004, the European Parliament created processes for the central authorization and supervision of medicines, including biologics. This regulation also allowed for clinical data to be omitted in the case of exceptional circumstances. A legal basis for biosimilars was created in 2005, which described the requirements for the market authorization application dependent on the scientific and clinical data demonstrating similarity to another biological medicinal product. The European framework recognizes that biosimilars demonstrate similarity but they are not identical to their reference products, given the complex protein structure, manufacturing process and final product differences. Furthermore, there is recognition that some product differences may be undetected for some time and may be difficult to understand. The European framework is supported by scientific guidelines and procedural guidance and implemented by the Committee for Medicinal Products for Human Use. This committee reviews marketing authorization applications, including those for biosimilars, and makes recommendations regarding approval to the European Commission (which formally approves medicinal products).

The EMA recognizes it is not economical to require a full common technical document for biosimilars and thus relies on a comparability exercise to reduce the amount of data required in Modules 4 and 5. The EMA has issued several guidelines on the comparability of pre- and post-manufacturing change for a given biologic drug. These guidelines have been broadened to include comparability of biologics by different manufacturers (biosimilars) and cover quality, clinical and non-clinical issues.

Additionally, biological class-specific guidelines have been developed to inform preclinical (PD and toxicological) and clinical (PD, PK, safety and efficacy) studies. The guidelines are subject to periodic revision as has been seen in 2008 with the addition of the recombinant erythropoietin agent guideline. More recently in 2013, a guideline introduced by the EMA acknowledges that for a structurally more “simple” biologic that can be very well characterized, an extensive clinical programme may not be necessary since the conclusion of biosimilarity may already be convincingly derived from the comparison of structural and functional characteristics and PK/PD studies (i.e. no remaining uncertainties). However, this would still not be a purely “generic drugs” approach because the extensive head-to-head comparison of the physicochemical and functional characteristics required for biosimilars is not part of the generic development programme. Similarity to the reference is important but the clinical benefit is considered proven with the reference product data. The revision also addresses the possibility of generic status for some biologics [17]. Finally, the EMA is considering a revision to the guideline regarding quality as it recognizes that the results of the initial comparability exercise may not hold true throughout the product’s lifecycle.

Fifteen biosimilar products are marketed in Europe. Of interest to this report, five ESA biosimilars have been available since 2007. Three are produced in one facility in southern Germany while two are produced in a northern German plant. Other biosimilars, namely somatropin and filgrastim, are also available in Europe [18]. Of note, epoetin theta (Biopoin® and Eporatio®) is not licensed as a biosimilar but as a new product supported by a full application.

### United States Food and Drug Agency

In the United States, SEBs are referred to as follow-on biological medications and in 2009 the FDA released a set of draft guidance documents to reflect the country’s current position on this topic [19-22]. Although similar to the EMA, WHO and Health Canada in many respects, the US is unique because the approval pathway for a follow-on biological medication depends on the type of application governing approval of the originator product, which is either a new drug application or a biologics licence application.

In 2010, the *Biologics Price Competition and Innovation Act* created a conceptually similar regulatory framework to the *Hatch-Waxman Act* allowing for an abbreviated process to approve follow-on biologics. Unlike a novel biologic that must be submitted through the biologics licence application pathway, an abbreviated application for follow-on biologics can be submitted under section 351(k) in the *Public Health Service Act.* For a 351(k) application to be successful, a clinical study or studies are required to demonstrate safety, purity and potency for one or more appropriate conditions of use for which the reference biologic is licensed. Like Health Canada, the indications for which a follow-on biologic is approved does not necessarily include all of the licensed uses of the innovator reference biologic but could vary depending on each biosimilar sponsor’s application and the extent to which the clinical trial information supports extrapolation across multiple indications.

Unlike Health Canada, the FDA believes that follow-on biologics and the reference biologic can be interchangeable (or substitutable according to Health Canada’s definition), meaning a pharmacist can switch to the follow-on biologic without the intervention of the prescriber. To achieve this status, the FDA requires follow-on biologic sponsors to demonstrate biosimilarity, that the follow-on biologic can be expected to produce the same clinical result as the reference product in any given patient and if the follow-on biologic is administered more than once to an individual, the risk in terms of safety or diminished efficacy of alternating or switching between the follow-on and the reference product is no greater than the risk of using the reference product without such alternation [22]. With regards to pharmacovigilance, the FDA acknowledges that robust post marketing safety monitoring is important but gives minimal information of what this entails, other than it should be product-specific. The FDA encourages sponsors to consult with the FDA to discuss their pharmacovigilance approach [20].

### Australian Therapeutic Goods Administration and the Japanese Pharmaceuticals and Medical Devices Agency

Both the Australian Therapeutics Goods Administration and the Japanese Pharmaceuticals and Medical Devices Agency (PMDA) have released their own guidelines for SEBs, which are virtually identical to the EMA regulatory framework [23-26]. Having a population of just over 23 million, Australia is an even smaller market than Canada [24,27]. Despite this, their early and swift adoption of the EMA guidelines without alteration provided a robust licensing system that has resulted in the marketing of SEBs such as epoetin lambda (HX575), causing a significant price competition in the treatment of anemia of CKD [24]. Australia has decided to distinguish a biosimilar from its reference product with regards to naming; hence, the biosimilar for epoetin alpha becomes epoetin lambda to indicate a difference in its glycosylation [28]. Like Australia, Japan has also commercialized HX575, but has allowed it to use the same INN with an extension, i.e. epoetin alpha BS (BS for biosimilar). In the first 12-months of its introduction, this SEB was estimated to have taken a quarter of the Japanese market share [29]. Since Japan is currently the world’s second largest pharmaceutical market after the United States, it is important to realize the significance of harmonizing its regulatory process with the EMA [30]. Until recently, one slight difference from the EMA was that the PMDA did not consider low molecular weight heparins as SEBs [31]. Therefore, there have been a number of low molecular weight heparins approved for use as generics [31].

### “Pharmemerging” countries – China, India and Thailand

China, India and Thailand are all countries with large populations within whom only a small minority are insured or have the economic ability to afford standard medical care. With increasing government expenditure on healthcare and a disease burden that has shifted from infectious diseases to chronic diseases, regulators in these countries are faced with even more pressure than those in Canada, the United States and Europe to facilitate the commercialization of SEBs. Currently, China and Thailand have not created an official SEB approval pathway. In Thailand, however, 14 different epoetin SEBs are marketed and all were approved using the regulatory process for chemical generics with no comparator studies to the originators [32]. Some argue that without demonstrating biosimilarity, these products cannot be considered true SEBs. In China, the State Food and Drug Administration (SFDA) approves all biologics as novel agents so that even though there are products on the market with the same generic name and approved for the same indication as the originator biologic, they have lower activity, less purity and do not meet international standards [33]. At the moment, China has minimal expertise with biologic development and a lack of facilities that are compliant with international Good Manufacturing Practices guidelines. However, this is rapidly changing with the increasing number of Western educated returnees with experience in large biotech companies, increasing China to become a global player with SEBs. Since China and Thailand do not have an SEB framework, pharmacovigilance for non-innovator biologics depends on existing drug surveillance systems. In China, surveillance systems have developed rapidly through partnership with the WHO and the pharmaceutical companies [30]. All ADRs are reported to The National Center for ADR Monitoring that consists of five divisions and a network of 32 provincial centers [30].Thailand is unique in that all new drugs must undergo a mandatory safety-monitoring period of approximately two years where only physicians in hospitals and clinics can prescribe the medications and only hospital and clinic pharmacies can dispense them [30]. It is encouraged that ADRs are reported to the Health Product Vigilance Center under the Thailand FDA [30].

Unlike China and Thailand, India’s regulatory framework has evolved quickly as a result of the country’s robust biotechnology sector of over 100 companies that are actively engaged in the development or production of copy biotherapeutic products [34]. By volume, India is the world’s 2^nd^ largest supplier of vaccines and the 4^th^ largest supplier of pharmaceuticals. As of 2011, the country had over 16 different non-innovator epoetins, one non-innovator rituximab and one non-innovator alteplase [34]. At the time these products were approved, no comparator studies were required and thus the commercialization of these products was relatively easy. This resulted in innovator price drops of 30 to 50%, sometimes two to three years ahead of a launch of a non-innovator product [34]. However in 2012, the country undertook major regulatory reform to align with EMA standards with the Department of Biotechnology and the Central Drugs Standard Control Organization releasing an SEB guidance document outlining regulatory requirements for marketing authorization [35]. Since then, to demonstrate biosimilarity, comprehensive product characterization, preclinical studies and clinical studies in comparison with a reference biologic must be undertaken [35]. Unlike Canada, SEBs can only be developed against an authorized reference biologic that has been approved in India and has been licensed and marketed for at least four years with significant safety and efficacy data. This can be waived or reduced in the case where no medicine exists, only palliative therapy is available or in the face of a national healthcare emergency [35].

Since India has adopted SEB guidelines similar to the EMA, a fairly vigorous pharmacovigilance plan is now mandatory for manufacturers in the post-marketing phase. This includes the submission of periodic safety update reports every six months for the first two years and then annually for another two years; in addition, at least one non-comparative post-marketing clinical study focusing on safety and immunogenicity must be conducted [35]. If any serious unexpected adverse reactions occur, they must be reported to the licensing authority within 15 days of initial receipt of the information [35].

## Predicting the potential Canadian market

Despite early optimistic projections of biosimilar market predictions, worldwide biosimilar sales in 2010 were $235 million of the $138 billion biologic market [36]. In the EU, only 8% of the 2.3 billion dollar market was captured by biosimilars [4]. Moreover, France and Germany account for half of the EU biosimilar market. The introduction of the monoclonal antibody infliximab biosimilar marks a significant advancement into large, complex proteins. In January 2014, Remsima™ (infliximab biosimilar, Celltrion Inc.), the brand name of infliximab biosimilar is set to be priced 39% less than the reference brand in Norway [37]. Within the EU, there are significant differences in market forces and hence biosimilar uptake. Moreover, biosimilars have had significant inter-country price variability, which affects comparisons. In 2009, for instance, Ratiopharm’s filgrastim biosimilar in Germany was 2.5 times its price in Spain [38]. Based on our analysis, under market phenomena similar to those seen in the EU, we could expect that Canadian adoption of epoetin SEBs would result in $35 million (year 1) to $50 million (year 5) cost savings annually, with cumulative savings of $221 million after 5 years. Below are some factors influencing price.

### Payor responses

It is useful to examine these differences and determine which country’s market, summarized in Table 2 will most resemble Canada in the years to come. The uptake of biosimilars seems to be greatest where the payer has greater influence, such as in Germany, compared to where physician influence through prescribing and brand loyalty dominate, such as in France, Italy and Spain [39]*.* Also worthy of note is that Spain and Italy were late adopters of biosimilars which may also explain a lower market share [29]. The class of biosimilars is also important. Agents typically used short term (such as filgrastim) are likely to be associated with greater biosimilar usage, while agents used long-term (such as long-term use of a growth hormone in a pediatric population) is associated with slow market growth [29].

**Table 2:** **Percent (%) biosimilar share of reference product sales** [14,39]

	**Percent (%) biosimilar share of reference product sales**								
	**Austria**	**France**	**Germany**	**Italy**	**Poland**	**Spain**	**Sweden**	**UK**	**EU total**
**Epoetin**	50	11	65	7	62	16	63	9	18
**Filgrastim**	52	42	45	18	38	24	45	80	38
**Somatropin**	6	20	12	12	7	15	21	4	13
**Infliximab**	**Approved but not yet marketed**								

Germany, as an early, rapid adopter, also has the highest percentage of epoetin biosimilar sales in Europe. In the first year, 17.3% or 60 million Euros were saved with epoetin biosimilar use. Germany is considered to have a “mature market” for the current biosimilar offerings and thus may have maximized their market share (65% for epoetin biosimilars). Several factors worth mentioning have played a role:

- Predefined quotas: In some areas in Germany there are quotas for biosimilar prescriptions that must be met. For epoetin, biosimilar prescriptions must be at least of 20% and 40% of the total in Hamburg and Berlin, respectively. Physicians may face fines for not meeting prescription quotas. This policy was a driver in the initial years, as in most areas today the biosimilar share exceeds these quotas. Canada does not have such measures [38].
- System of reimbursement: In Germany, doctors and hospitals are paid based on diagnostic related groups so regardless of treatment, funding is the same amount per patient per disease. The incentive to use biologics that are 30% cheaper is obvious to prescribers and providers. The central Federal Healthcare sets reimbursement rates which favours biosimilar use. While Canada does monitor costs per diagnostic related groups and encourages lower costs, there is no direct penalty; however the principle of efficient use of healthcare dollars is promoted in Canada.
- Reference pricing: Germany has a reference-based pricing system in which the cheapest generic price is set as the reference price. As cheaper generics are introduced, the reference price falls. Furthermore, a recent law allows biosimilar manufacturers, providers and hospitals to negotiate rebates directly. Hence, there are many health insurance funds that have a major influence on medications patients receive. The net effect has been to have the same reference price for a given biologic including its biosimilars. In 2011, Germany introduced cost benefit legislation in which drugs that have been deemed to have additional value can have a negotiated price with health insurance funds while those that do not will be subject to the reference based price. Going forward, biosimilars, by definition, will be subject to a reference price [40].

In Poland, annual national tendering with pharmaceutical companies is common and has the effect of switching whole patient groups from one somatropin to another and then back again. This large scale interchange of a biologic in many EU countries (such as Germany, Sweden, Spain and Netherlands) is forbidden but in Poland, there have been no signals of problems identified including immunogenicity [41]. As a result of this tendering process, selection is price sensitive and has favoured SEBs as is evidenced by the market penetration.

### Market responses

#### Expected price reductions and influence on overall costs

While a generic drug may cost 2 to 3 million dollars to bring to market, the estimated cost for an SEB is 75 to 250 million dollars, depending on the requirements for clinical studies and analysis, making deep discounting unlikely [42,43]. Information from Europe suggests that SEBs will be priced 25 to 30% lower than the innovator biologic drug. Average price reductions are 23% in the EU, and 30% in Japan [38]. In Canada, large buying groups or organizations typically purchase innovator biologics below list price in the form of institutional pricing or value added contracts, which may include rebates, additional services or offerings by the vendor. As such, the actual price difference will be less than a 25% difference from list. More recently, two biosimilar infliximabs approved in Europe in September 2013 and in Canada in January 2014, are anticipated to be around 30% less than list price.

Manufacturers of innovative biologics have responded in Europe by reducing prices. For example, Germany enjoyed a 13% price reduction for the innovator epoetin [38,40]. Likewise, Roche reduced prices in India to compete with biosimilar products there. Moreover, not only did the reference product price drop, but prices for second and third generation erythropoietic agents fell as well. These competitive pricing changes have, in part, been responsible for a slow market penetration of biosimilars. Hence, whether the SEB is used or not, the overall costs for the class of medications will be reduced.

Both the number of SEBs and SEB uptake may affect the reference product price. The 2013 price of the SEB epoetin and the reference drug is now only 1% different in Germany – a country with rapid and early adoption of epoetin SEBs and with four epoetins on the market. In Spain, a relative newcomer to epoetin SEBs and with only two epoetins on the market, there is a 30% price differential. Italy maintains a high price (73% higher than in Germany) for the reference epoetin with the SEBs priced just 15% less than the reference. Tables 3 and 4 compare five European countries’ epoetin data comparing 2009 with 2013. Of note, the British Pound gained in value against the Euro, making the price comparatively low.

**Table 3:** **2009 Price comparison and market share of SEB Epoetins in five European countries** [38,40]

	**Germany**	**UK**	**France**	**Italy**	**Spain**
**Year of 1** ^**st**^ **SEB introduced**	2007	2008	2008	2008	2009
**Number of SEB epoetins on market**	4	1	1	3	2
**SEB market share (sales as%)**	53	0.95	2.97	0.29	2.91
Price innovator vs SEB (€/ddd)*	9.3 vs. 6.5	9.5 vs. 7.8	11.4 vs. 7.4	9.5 vs.7.8	8.5 vs. 6.5
**Average price difference between innovator epoetin and SEB (%)**	43	22	54	22	32

*€/ddd = Euros per defined daily dose. The WHO has defined the ddd for Epoetin to be 1,000 international units per day.

**Table 4:** **2013 Pricing comparison of SEB Epoetins in five European countries** [44]

	**Germany**	**UK**	**France**	**Italy**	**Spain**
**Number of SEB epoetins on market**	4	2	2	3	2
Price innovator vs SEB (€/ddd)*	6.06 vs. 6.02	5.65 vs. 5.20	6.65 vs. 5.72	10.47 vs 8.91	8.65 vs. 6.06
**Average price difference between innovator epoetin and SEB (%)**	1	8	14	15	30

*€/ddd = Euros per defined daily dose. The WHO has defined the ddd for Epoetin to be 1,000 international units per day.

#### Likely subsequent entry biologic companies entry into the Canadian market

It is possible that only established SEB companies with a track record in Europe or other highly regulated countries will bring SEBs to Canada. As such, they will use their considerable experience to influence their success. Price will be a very important factor, but companies may leverage the safety and efficacy data from years of use elsewhere. In some jurisdictions in Canada, value-added incentives from innovator companies are important to the operation of renal programs. In these areas, SEB manufacturers may need to offer such incentives to be successful but this may affect ultimate pricing or market penetration.

#### Expected response in Canada to SEBs by innovator companies

**Figure 1:**
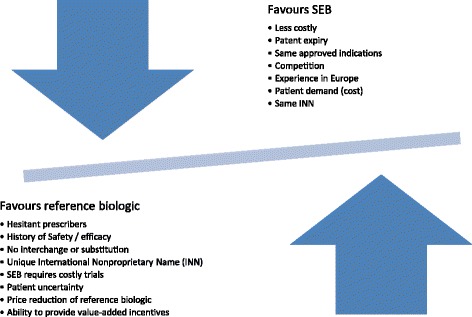
**Factors balancing the use of an innovator biologic vs. an SEB.**

Experience elsewhere demonstrates that companies will employ several strategies to retain market share. Patent challenges and other legal manoeuvres will be an initial line of defence and may well delay the SEB or result in licensing fees paid to the innovator. Secondly, they may develop persuasive marketing messages on subjects like preventing substitution (a message already supported by Health Canada) [45]. In the United States, innovator biologic companies may market directly to the consumer and may highlight the differences in the totality of evidence between innovator and SEBs; some of this may spillover into Canada. They may also lobby government and regulators as well as relying on strong trusted branding. Finally, and perhaps most persuasively, innovator companies will likely lower their price to match or be close to the SEB price, as in Germany. For ESAs, this may also affect the price of darbepoetin. Figure 1 illustrates the factors that may favour either the innovator or SEB market.

## Implications in Canadian nephrology practice

As part of this research, we approached several pharmaceutical manufacturers with molecules of interest in nephrology and asked if they were aware of any SEB applications with Health Canada for Eprex® (epoetin alfa, Janssen Inc.), Aranesp® (darbepoetin alpha, Amgen Inc.), Cathflo® (alteplase, Hoffmann-La Roche Ltd.) or Rituxan® (rituximab, Hoffmann-La Roche Ltd.). Each company responded but only one indicated it was aware of any applications. Hospira and Sandoz have each filed applications for epoetin SEBs with Health Canada.

### Timelines for SEB entry

#### Epoetin SEB

Five epoetin biosimilars were introduced to Europe in 2007, just a few years after the reasons for the spike in PRCA cases were elucidated [36,46,47]. With this backdrop and with Europe being the first industrialized region to introduce epoetin biosimilars, it is understandable that medical professionals expressed concern about the safety of biosimilars with respect to immune responses. Physicians had to weigh that uncertainty against the possible cost savings [48,49]. Indeed, when Eprex® could be again administered by subcutaneous route, the biosimilars were evaluated for this route as well. In 2009, a study involving the epoetin biosimilar HX575 was halted after two patients developed neutralizing anti-erythropoietin antibodies [8]. In 2010, another epoetin biosimilar (SB309) did gain approval for the subcutaneous route of administration. The EMA Biosimilar Recombinant Erythropoietin Guideline was revised in 2010 to address these various concerns and specifically state subcutaneous testing must be performed. As the patent expired for Eprex® on May 27, 2014, we predict Health Canada will encounter two epoetin SEBs in either 2014 or 2015.

#### Darbepoetin SEB

Like rituximab, India has an intended darbepoetin biosimilar approved but it should not be regarded as a true biosimilar. However, there are no indications that companies are currently creating a darbepoetin SEB elsewhere. The authors do not anticipate a darbepoetin SEB in Canada in the next five years. That being said, it is possible that the price of Aranesp® may be lowered in response to an epoetin SEB.

#### Rituximab SEB

Since April 2007, Dr. Reddy’s Laboratories (Hyderabad, India) has marketed Reditux™, an intended rituximab biosimilar in India, Bolivia, Chile and Peru [50]. Reditux™ should not be regarded as a true SEB and thus cannot be licensed in the EU or North America according to the current biosimilar regulations since it was introduced prior to the development of biosimilar regulatory pathways and did not need to demonstrate biosimilarity [50]. Probiomed (Mexico City, Mexico) has marketed Kikuzubam®, another intended rituximab biosimilar, in Mexico, Bolivia, Chile and Peru. The Mexican Ministry of Health approved Kikuzubam® before regulations regarding approval of biosimilars had been implemented on April, 20^th^ 2012 [50]. Aside from Dr. Reddy’s Labs and Probiomed, Boehringer Ingelheim, Sandoz, Celltrion (Korea), Samsung biologics (Korea), Teva/Lonza (Italy), Pfizer and Merck are all developing a rituximab SEB. Pfizer is co-developing a rituximab SEB and has entered phase I and phase II trials. No rituximab SEB will be available in Canada within the next 5 years.

#### Alteplase SEB

Lastly like rituximab and darbepoetin, there is an intended alteplase biosimilar marketed in China but there are no indications of any other alteplase biosimilar in development and thus the authors do not anticipate an SEB entering the Canadian market within five years.

### Provincial and federal influences

#### Interchangeability

Interchangeability is a clinical decision in which one drug is changed to another drug within the same class. A product is interchangeable with another if both products are approved for the same indication. A common example would be the interchange of an ACE inhibitor from trandolapril to ramipril. This decision typically rests with a medical committee responsible for a formulary. Health Canada advises that the notice of compliance does not indicate therapeutic or pharmaceutical equivalence [14]. Interchangeability between molecules, however, is a provincial decision and at the local level can be agreed upon by an organization’s medical advisory committee; Health Canada recommends that physicians make only well-informed decisions regarding this [14]. As in Italy where individual health regions have decided to interchange a SEB with the original biologic, provinces, health authorities and hospitals in Canada may elect to follow suit. It is important to understand that interchangeability does not imply substitutability.

A policy decision to interchange an originator biologic with an SEB would have to be made thoughtfully, keeping in mind that the characteristics of an SEB are different from the original and while similar, may result in a period of dose instability just as it could be when switching between two originator products. If the interchange was made for an entire population, the issues regarding maintaining two or more sets of patients on specific molecules would disappear, as would the local effort to discern between a biologic and its SEB(s). The population would be simply switched over and after a period of time, doses would stabilize and any adverse events would be reported for that molecule, since it would be the only one in use as new prescriptions would be interchanged for the SEB.

A formulary committee could also decide to only interchange in patients not previously exposed to the originator molecule. This phased-in approach would have the encumbrances of maintaining separate stocks, creating processes to ensure patients were not inadvertently switched to the other manufacturer’s molecule and give direction on how prescribers would write orders. A prescriber could no longer write “epo”, for instance, but would have to be clear on which product is intended such as by prescribing with the brand name. Encouraging brand names in prescriptions certainly goes against the direction of hospitals, organizations and societies that promote use of the international non-proprietary name (INN) [51]. To the regulator, the ability to trace which product a patient received is paramount to a good pharmacovigilance program. Operationally, it is also useful for the care team to have clear direction, which biologic is intended by the prescriber. Frequent switching between SEBs or between the innovator and SEB may introduce some variability in the observed outcomes and would make causality of an observed adverse reaction more difficult to assign. Additionally, after a period of time, the safety and efficacy of an SEB and the reference biologic may drift as each undergoes manufacturing changes. The magnitude of such a drift is unknown but remains a possibility and is another reason to be cautious about frequent switching.

#### Substitution

Substitution is described as substituting one drug for another that is bioequivalent. In Canada, this is typically seen with a brand name being substituted for a generic version. Substitution is regulated by the provincial Colleges of Pharmacy and provincial legislation. There is variability across the country with respect to which drugs can and cannot be substituted. When a substitution is made, no notification to the prescriber or health care team is necessary. Health Canada, however, does not support automatic substitution of an original biologic with an SEB. Several EU member countries have specific legislation regarding this: Spain, France, Italy and the United Kingdom have do not allow automatic substitution [38]. The French government passed legislation allowing pharmacists to substitute a biosimilar drug when a reference biologic is prescribed but only for patients who are new to that biologic [52].Germany only allows substitution between biosimilars if the two brands of biosimilars originate from the same manufacturing plant (as is the case with the epoetins). Norway had introduced legislation to mandate substitution from innovator to biosimilar but the courts subsequently struck this down, as biosimilars are not bioequivalent. Mexico allows substitution of drugs with the same INN, which includes biologics. Japan has stated substitution should be avoided in the post-marketing phase.

Health Canada’s position is that SEBs are not automatically substitutable and advises against it at this time. Alberta has decided biologics are not substitutable or interchangeable while BC leaves this decision to the individual pharmacist. Other provincial regulators have not made any decisions.

Unfortunately, the terms “interchangeable” and “substitution” are used interchangeably in Canada and this will need to change in the era of SEBs.

#### Third party payer decisions

Provincial public drug plans and private health insurers may also influence the uptake of SEBs in Canada. Introducing policies to therapeutically interchange innovator biologics with SEBs would have an impact (such as SEB-first in biologic naive patients for short term filgastrim use). Policies introduced to fund therapy up to the cost of an SEB would place pressure on both prescribers and patients that would encourage use of SEBs.

For the molecules of interest to nephrology, the landscape in Canada is one of a government payer funding the drug, and as such third party payers may have a lesser impact. Currently there is little or no cost to patients for ESAs or alteplase. Rituximab funding is variable, but is often administered in a hospital setting, and may be subject to “SEB first” policies.

A phased-in approach might be considered, that is, after a period of time during which prescribers feel confident in the SEBs, a regulator or drug plan administrator may entertain methods to switch patients to the cheaper alternative. Until such time, the authors believe that the decision to use an SEB should rest with the prescriber and patient.

#### Influence of patient advocacy groups

In Canada, many chronic disease states have patient advocacy groups. For a number of groups, biologics have provided significant benefits to the patients. Given the cost of these agents, there is considerable focus on improving patient access both earlier in the disease course but also to remove cost as a barrier to patients in scenarios where it is not readily available. Alternatively, advocacy groups may wish to maintain the status quo if they perceive uncertainties with SEBs. Within nephrology practice, the biologics of concern with respect to timely access are rituximab and eculizumab. Neither product will have an SEB in the medium term.

#### Influence of buying groups

Group-purchasing organizations (GPO) contract prices are negotiated with pharmaceutical companies and the lowest price is usually granted a contract or attracts the most purchases. Exclusive contracts may offer greater discounts or incentives. In the realm of original biologic manufacturers competing with an SEB manufacturer, several scenarios may develop. Should the GPO favour one SEB over another, that SEB will become the SEB of choice for members of the GPO (hospitals, health authorities, agencies and pharmacy chains). With respect to pharmacovigilance and mitigating the risk of patients being exposed to multiple SEBs, this may be a desired outcome. However, as is the nature of contracts, the next contract may prefer a different brand of SEB and this may evoke a large-scale switch from one SEB to another, and may require close monitoring for a period of time to assess for differences in efficacy. GPOs must develop strategies to address SEBs in the long term to create the right balance between patient safety and best price. Within Canadian nephrology, entire provinces favour one ESA over another so the concept is not new, but has not yet been applied to SEBs.

#### Purchasing targets

Provincial governments may welcome SEBs as a strategy to reduce the growth of health expenditure while providing the same level of care. In order to support already expensive health care systems, governments may dictate that specific percentages of biologic purchases must be for SEBs. This strategy could prove quite effective in achieving spending targets but may place practitioners in positions to prescribe every de novo patient an SEB. Further, the target may give cause to switch stabilized patients to an SEB. Regions in Germany have such a policy, which not only help to reduce cost, but also supports the German SEB industry.

In “pharmemerging” countries where governments are less involved in funding expensive biologics, there is a faster and greater patient and prescriber acceptance of SEBs. Hence, purchasing targets become unnecessary.

### Educational issues

Physicians and health care providers need to have at least a basic understanding of the science of the comparability exercises, the theoretical risks with using SEBs, the guidelines regulating their use and pharmacovigilance programs. Additionally, a clear understanding of the interchangeability and substitution rules in their jurisdiction is essential as there initially may be uncertainty in the local and provincial rules.

Patients must also be educated about the science behind SEBs and their pros and cons prior to being prescribed or switched to an SEB. This education should come from their physician and pharmacist and not solely from the pharmaceutical industry.

## Recommendations

After extensive research into the area, it is clear that we are in the infancy stages in our understanding of SEBs and their impact on patients and the health care system. For those who may be interested, the authors have created a set of opinion-based recommendations to serve as a starting point for discussing SEB use in nephrology practice. These points should be used for contemplation and not be taken as guidelines. As experience grows and new information becomes available, these viewpoints may evolve or change.

### Education

- Patients and clinicians should consider the use of subsequent entry biologics but only after fully understanding their unique challenges and opportunities through unbiased education.
- Education on subsequent entry biologics is the responsibility of clinicians and government health agencies and should not rest solely with the pharmaceutical industry.

### Clinicians

- Clinicians should advocate that subsequent entry biologics not be automatically substitutable. Therapeutic interchange should be a well-informed, decision that involves a physician, pharmacist, patient or medical committee when switching populations stabilized on the originator.
- Physicians should prescribe the originator biologic and its subsequent entry biologics in such a manner that is clear as to which product is intended (e.g. by brand name if necessary) since these products are not substitutable.
- Patients not previously exposed to, or stabilized on an innovator biologic should be potential candidates for subsequent entry biologics.
- It is preferable if subsequent entry biologics are only used for Health Canada-approved indications as these indications are granted only if sufficient data is provided. Clinicians should be vigilant when using subsequent entry biologics and be willing to file adverse event reports to Health Canada for any suspected untoward event – either for the subsequent entry biologic or the original biologic. Only through good pharmacovigilence will health care providers and patients become better informed of the similarities or differences between agents.

### Policy and decision makers

- Key policy decisions should take into consideration physician, pharmacist and patient preferences such as those that can be elicited through discrete choice experiments, in addition to the financial variables.
- Should a subsequent entry biologic be launched in Canada that has European experience and originates from the same facility, preference should be given to that product over others with less experience.
- For the erythropoiesis stimulating agents, a standardized anemia management protocol (used in many settings) for both subsequent entry biologics and originator biologics would enable real world comparisons. Under regularly monitored conditions such as this, switching entire populations such as a dialysis unit to a subsequent entry biologic could be considered.
- Nephrology should work with rheumatology, oncology, gastroenterology and other providers with regards to subsequent entry biologic policies since the use of these drugs will impact all patients. A tiered system where one set of patients receives an originator biologic and another group does not may create conflict and confusion in the Canadian health care system.
- Hospitals in particular will have considerable leverage to negotiate reduced pricing on the originator biologic after a subsequent biologic comes to the market. However, hospitals should consider who the majority payer will be and make decisions with a global perspective. For example, if a patient were started on an originator biologic while in hospital, the probability of switching to an SEB after discharge is slim.
- Decision makers should use the flexible pharmacoeconomic models described in the full report to generate anticipated costs using the real inputs particular to their environment. This will assist in the decision to continue with the originator biologic or move to a subsequent entry biologic.

## Conclusion

Subsequent entry biologics are an opportunity as well as a challenge for the Canadian population. This report summarized the international experience with subsequent entry biologics, and the Canadian perspective on this challenge. It compares Canadian regulations to other developed nations, discusses clinical issues with the use of subsequent entry biologics and it predicts their impact on the Canadian market and on nephrology practice. After extensive research in the rapidly evolving field of subsequent entry biologics, it is clear that we are only beginning to understand the impact that subsequent entry biologics will have on Canadian nephrology practice. We hope that this report will help clinicians and policy makers to navigate this complex subject and to make informed decisions in the best interest of their patients.
